# Association Between COVID-19 Vaccination and Artificial Insemination Outcomes for Couples Experiencing Infertility

**DOI:** 10.1001/jamanetworkopen.2022.47216

**Published:** 2022-12-16

**Authors:** Chao Wang, Dongdong Tang, Jiayin Liu, Songying Zhang, Yanwen Xu, Jie Qiao, Yunxia Cao

**Affiliations:** 1 Department of Obstetrics and Gynecology, First Affiliated Hospital of Anhui Medical University, Hefei, China; 2 National Health Commission Key Laboratory of Study on Abnormal Gametes and Reproductive Tract, Anhui Medical University, Hefei, China; 3 Key Laboratory of Population Health Across Life Cycle, Anhui Medical University, Ministry of Education of the People’s Republic of China, Hefei, China; 4 Clinical Center for Reproductive Medicine, First Affiliated Hospital of Nanjing Medical University, Nanjing, China; 5 Assisted Reproduction Unit, Department of Obstetrics and Gynecology, Sir Run Shaw Hospital, Zhejiang University School of Medicine, Hangzhou, China; 6 Reproductive Medical Center, First Affiliated Hospital of Sun Yat-sen University, Guangzhou, China; 7 Center for Reproductive Medicine, Peking University Third Hospital, Beijing, China

## Abstract

This cohort study investigates the association between COVID-19 vaccination status and artificial insemination by partner outcomes among couples experiencing infertility in China.

## Introduction

China has used 3 types of vaccines to prevent COVID-19: inactivated, adenovirus, and recombinant vaccines. However, many couples experiencing infertility have hesitated to undergo vaccination.^1^ Misinformation claiming that the COVID-19 vaccine causes female infertility has circulated on social media.^2,3^ Although some studies^4,5,6^ have focused on the association of vaccines with in vitro fertilization outcomes, no evidence involving artificial insemination by partner (AIP) has been reported, to our knowledge. This multicenter prospective study investigated the association of COVID-19 vaccination with AIP outcomes.

## Methods

This cohort study was approved by the Research Ethics Board of the First Affiliated Hospital of Anhui Medical University, and participants signed informed consent at the start of treatment. This study followed the Strengthening the Reporting of Observational Studies in Epidemiology (STROBE) reporting guideline. We collected clinical data for 4185 couples who received their first AIP treatment from 10 centers in 9 provinces in China from July 2021 to February 2022.

Before AIP treatment, we registered vaccination information and demographic characteristics of couples. Transvaginal ultrasonography was conducted to detect clinical pregnancy 1 month after AIP, which could show the presence of a gestational sac in the uterus.

Demographic characteristics and variables were summarized using descriptive statistics, including means with SDs and frequency counts with percentages. Multivariate regression analysis was used to derive adjusted odds ratios (aORs) and 95% CIs for the association between vaccination status and odds of clinical pregnancy. Confounders considered were variables with *P* values < .25 in univariate analysis. A 2-tailed *P* value <. 05 was considered statistically significant. Data analysis was performed using SPSS statistical software version 22.0 (IBM).

## Results

The study group consisted of 4185 couples who received their first AIP treatment after COVID-19 vaccination, including 3582 couples without and 603 couples with clinical pregnancies. The groups had similar characteristics at baseline, except mean (SD) age (31.49 [3.70] years vs 30.66 [3.86] years) and body mass index (calculated as weight in kilograms divided by height in meters squared; 21.81 [3.23] vs 22.16 [3.21]) (Table 1). (These measures were for the women in the couples only.)

**Table 1.** Characteristics of Participants

Characteristic	Clinical pregnancy, No. (%) (N = 4185)		*t*/χ^2^	*P* value
	No (n = 3582)	Yes (n = 603)		
Age, mean (SD), y^a^	31.49 (3.70)	30.66 (3.86)	5.109	<.001
Infertility duration, mean (SD), y^a^	2.67 (2.01)	2.56 (1.93)	1.286	.20
BMI, mean (SD)^a^	21.81 (3.23)	22.16 (3.21)	−2.380	.02
FSH, mean (SD), mIU/mL^a^	7.04 (2.35)	6.89 (2.45)	1.395	.16
Type of infertility^a^				
Primary	2590 (72.3)	438 (72.6)	0.028	.87
Secondary	992 (27.7)	165 (27.4)		
Type of cycle				
Natural	1202 (33.6)	191 (31.7)	2.423	.30
Medication	1702 (47.5)	307 (50.9)		
Unknown	678 (18.9)	105 (17.4)		
Woman				
Vaccination status				
Vaccinated	1363 (38.1)	218 (36.2)	0.792	.37
Unvaccinated	2219 (61.9)	385 (63.8)		
Vaccine type				
Adenovirus	23 (1.7)	3 (1.4)	0.490	.81^b^
Inactivated	1302 (95.5)	211 (96.8)		
Recombinant	38 (2.8)	4 (1.8)		
Time interval				
First vaccination to AIP, mean (SD), d	146.34 (63.86)	153.79 (67.59)	−1.585	.11
Last vaccination to AIP, mean (SD), d	119.31 (61.63)	124.74 (63.21)	−1.204	.23
Man				
Vaccination status				
Vaccinated	1697 (47.4)	266 (44.1)	2.207	.14
Unvaccinated	1885 (52.6)	337 (55.9)		
Vaccine type				
Adenovirus	39 (2.3)	6 (2.3)	0.195	.91
Inactivated	1598 (94.2)	252 (94.7)		
Recombinant	60 (3.5)	8 (3.0)		
Time interval				
First vaccination to AIP, mean (SD), d	153.51 (68.08)	155.01 (67.14)	−0.334	.74
Last vaccination to AIP, mean (SD), d	125.10 (65.97)	124.64 (63.55)	0.105	.92
Both partners				
Vaccination status				
None	1746 (48.7)	310 (51.4)	2.393	.49
Only woman vaccinated	139 (3.9)	27 (4.5)		
Only man vaccinated	473 (13.2)	75 (12.4)		
Both vaccinated	1224 (34.2)	191 (31.7)		

Abbreviations: AIP, artificial insemination by partner; BMI, body mass index (calculated as weight in kilograms divided by height in meters squared); FSH, follicle stimulating hormone.

SI conversion factor: To convert milli-international units per milliliter to international units per liter, multiply by 1.0.

^a^ Measure is for woman in a couple only.

^b^ Fisher exact test.

In multivariate regression analysis, the woman’s vaccination status was not associated with odds of AIP pregnancy (aOR, 1.008; 95% CI, 0.786-1.293). In cycles with all women vaccinated, the vaccination of the woman with adenovirus, inactivated, or recombinant vaccine was not associated with odds of pregnancy. Moreover, the times from the woman's first and last vaccination date to AIP operation were not associated odds of pregnancy (Table 2).

**Table 2.** Association of COVID-19 With Odds of Pregnancy

Vaccine variable	Pregnancy, aOR (95% CI)
Woman	
Vaccination status	
Unvaccinated	1 [Reference]
Vaccinated	1.008 (0.786-1.293)
Vaccine type	
Adenovirus	1 [Reference]
Inactivated	1.074 (0.313-3.687)
Recombinant	0.524 (0.095-2.896)
Time interval^a^	
First vaccination to AIP, d	1.005 (0.999-1.012)
Last vaccination to AIP, d	0.997 (0.990-1.004)
Man	
Vaccination status	
Unvaccinated	1 [Reference]
Vaccinated	0.810 (0.517-1.271)
Vaccine type	
Adenovirus	1 [Reference]
Inactivated	1.941 (0.449-8.394)
Recombinant	1.145 (0.199-6.588)
Time interval^a^	Time interval
First vaccination to AIP, d	0.998 (0.995-1.001)
Last vaccination to AIP, d	0.998 (0.995-1.001)
Both partners	
Vaccination status	
None	1 [Reference]
Only woman vaccinated	1.097 (0.708-1.700)
Only man vaccinated	0.917 (0.692-1.216)
Both vaccinated	0.890 (0.731-1.085)

Abbreviations: AIP, artificial insemination by partner; aOR, odds ratio.

^a^ Time intervals were associated with odds of pregnancy per unit increase in time.

Similarly, the man’s vaccination status was not associated with odds of pregnancy (aOR, 0.810; 95% CI, 0.517-1.271). Additionally, in regression analysis, type of vaccination (adenovirus, inactivated, or recombinant) and time from vaccination to AIP operation were not associated with odds of pregnancy (Table 2).

We divided couples into 4 categories by COVID-19 vaccination status: (1) 2056 couples with neither partner vaccinated, (2) 166 couples with only the woman vaccinated, (3) 548 couples with only the man vaccinated, and (4) 1415 couples with both partners vaccinated. Group membership was not associated with odds of pregnancy (Table 2).

## Discussion

This cross-sectional study has several limitations. There was 1 preovulatory or postovulatory AIP procedure performed in individual cycles, and this study did not distinguish between these parts of the cycle. Owing to the short duration of the study, associations with pregnancy outcomes and complications were not analyzed.

This multicenter prospective study found that the 3 COVID-19 vaccines used in China were not associated with lower odds of AIP pregnancy. These findings suggest that these vaccines should be used in China among couples planning pregnancy or AIP treatment and that couples need not delay pregnancy schedules because of COVID-19 vaccinations.

## Notes

Supplement.
Data Sharing Statement
