# Supplementary material for: Association Between COVID-19 Vaccination and Artificial Insemination Outcomes for Couples Experiencing Infertility
Source: JAMA Netw Open. 2022 Dec 16;5(12):e2247216. doi: 10.1001/jamanetworkopen.2022.47216 (PMC9856378; doi:10.1001/jamanetworkopen.2022.47216)
Supplement: Supplement. — Data Sharing Statement [file jamanetwopen-e2247216-s001.pdf]

Data Sharing Statement

Wang C, Tang D, Liu J, et al. Association between COVID-19 vaccination and artificial insemination outcomes for couples experiencing infertility. *JAMA Netw Open*. 2022;5(12):e2247216. doi:10.1001/jamanetworkopen.2022.47216

Data

Data available: Yes
Data types: Deidentified participant data, Participant data with identifiers, Data (not involving human participants), Data dictionary
How to access data: caoyunxia5972@ahmu.edu.cn
When available: With publication

Supporting Documents

Document types: Informed consent form
How to access documents: caoyunxia5972@ahmu.edu.cn
When available: With publication

Additional Information

Who can access the data: researchers whose proposed use of the data has been approved
Types of analyses: for any purpose or for a specified purpose
Mechanisms of data availability: with investigator support

© 2022 Wang C et al. *JAMA Network Open*.
